# Comparative Study on Iron Content Detection by Energy Spectral CT and MRI in MDS Patients

**DOI:** 10.3389/fonc.2021.646946

**Published:** 2021-03-22

**Authors:** Yao Zhang, Chao Xiao, Jing Li, Lu-xi Song, You-shan Zhao, Shuang Han, Zhao-wei Li, Cha Guo, Jun-gong Zhao, Chun-kang Chang

**Affiliations:** ^1^Department of Hematology, Sixth People's Hospital Affiliated to Shanghai Jiao Tong University, Shanghai, China; ^2^Department of Radiology, Sixth People's Hospital Affiliated to Shanghai Jiao Tong University, Shanghai, China

**Keywords:** MRI, DECT, ASF, iron overload, MDS

## Abstract

**Objective:** The purpose of this study was to identify the difference between dual energy spectral computed tomography (DECT) and magnetic resonance imaging (MRI) used to detect liver/cardiac iron content in Myelodysplastic syndrome (MDS) patients with differently adjusted serum ferritin (ASF) levels.

**Method:** Liver and cardiac iron content were detected by DECT and MRI. Patients were divided into different subgroups according to the level of ASF. The receiver operating characteristic curve (ROC) analysis was applied in each subgroup. The correlation between iron content detected by DECT/MRI and ASF was analyzed in each subgroup.

**Result:** ROC curves showed that liver virtual iron content (LVIC) *Az* was significantly less than liver iron concentration (LIC) *Az* in the subgroup with ASF < 1,000 ng/ml. There was no significant difference between LVIC *Az* and LIC *Az* in the subgroup with 1,000 ≤ ASF < 2,500 ng/ml and 2,500 ≤ ASF < 5,000 ng/ml. LVIC *Az* was significantly higher than LIC *Az* in the subgroup with ASF <5,000 and 5,000 ≤ ASF ng/ml. In patients undergoing DECT and MRI examination on the same day, ASF was significantly correlated with LVIC, whereas no significant correlation was observed between ASF and LIC. After removing the data of ASF > 5,000 mg/L in LIC, LIC became correlated with ASF. There was no significant difference between the subgroup with 2,500 ≤ ASF < 5,000 ng/ml and 5,000 ng/ml ≤ ASF in LIC expression. Furthermore, both LIC and liver VIC had significant correlations with ASF in patients with ASF < 2,500 ng/ml, while LVIC was still correlated with ASF, LIC was not correlated with ASF in patients with 2,500 ng/ml ≤ ASF. Moreover, neither cardiac VIC nor myocardial iron content (MIC) were correlated with ASF in these subgroups.

**Conclusion:** MRI and DECT were complementary to each other in liver iron detection. In MDS patients with high iron content, such as ASF ≥ 5,000 ng/ml, DECT was more reliable than the MRI in the assessment of iron content. But in patients with low iron content, such as ASF < 1,000 ng/ml, MRI is more reliable than DECT. Therefore, for the sake of more accurately evaluating the iron content, the appropriate detection method can be selected according to ASF.

## Introduction

MDS patients may suffer from iron overload as a result of a long-term transfusion of red blood cells (RBC) and/or combined with abnormal iron metabolism. The liver is the first organ to store excess iron and excess iron is also deposited in other organs including the heart and pancreas. Liver biopsy analysis is used as the reference standard for the measurement of liver iron concentration; however, it is difficult to implement in clinical practice, as there is an increase in bleeding risk. Serum ferritin (SF) is the most commonly used and convenient method to estimate iron stores, however, the results are easily affected by other factors (such as infection, inflammation, tumor, etc.) which sometimes mean they cannot reflect the amount of iron in the body. Since the previous study reveals that SF is positively correlated with CRP, we have employed the adjusted SF instead of SF for iron assessment (1).

The qualitative detection of iron using MRI is widely used in clinical practice and is considered an indicator for initiating iron chelation therapy (ICT). It is feasible to evaluate LIC and MIC by quantitative MR relaxation parameters (R2 and R2 ^*^) (2). However, in the case of extremely severe iron deposition, the MR signal attenuates rapidly, which can lead to a decrease of R2 ^*^ measurement accuracy (3). *In vitro* experiments show that DECT have higher accuracy in evaluating iron overload compared with 1.5T MRI (4). The virtual iron content (VIC) obtained from post-processing software is helpful to accurately evaluate the hepatic iron content. However, the sensitivity of VIC is significantly lower than that of R2^*^ when the LIC threshold is lower than 1.8 mg/g dry tissue iron concentration (5). In patients whose ferritin levels are close to the normal range, VIC values still fluctuate greatly (6, 7). At present, 3.0T MRI is widely used in clinical practice. What are the differences between 3.0T MRI and DECT in the detection of iron content? What are the advantages and disadvantages of these two methods in patients with high and low iron deposition? Can these two methods complement each other? This study hopes to answer these questions.

## Materials and Methods

This study was conducted in accordance with the Declaration of Helsinki and was approved by the Ethics Committee of our hospital. Written informed consent was obtained from all participants.

### Cases and Groupings

This retrospective data evaluation was approved by the ethics committee of our hospital (approval No. 2021-004). Patients were included in this study if they were suspected of having liver iron overload or an elevated ASF level (>500 ng/ml). Furthermore, we excluded cases with known liver pathology (active form of hepatitis, cirrhosis, status post liver transplantation, hepatic manifestations of underlying disease), combined with definite infection, cases in which the patient had received ICT during the previous 6 months and cases without corresponding ASF values. We retrospectively identified and assessed a consecutive series of 181 MDS patients between September 2014 and February 2020. MDS diagnosis was performed in accordance with the 2012 expert consensus (8). Typing was in accordance with the 2016 WHO classification standards (9). Iron overload diagnosis was in accordance with the 2011 standard (10). Among these, 146 MDS patients had a history of blood transfusion, and 77 patients had transfusion dependence. IPSS-R (11) and WPSS scores (12) were used for evaluation and grading. The low-risk group of patients were treated with cyclosporine, thalidomide, and other immunomodulatory and hematopoiesis promoting therapies, the medium and high-risk group was treated with demethylation or CHG pre-excitation of chemotherapy, and the leukemic transformation (tAML) group was treated with demethylation combined with myeloid regimen chemotherapy. Liver protection or anti heart failure therapy were performed if necessary during the treatment. Among them, six cases had hepatitis (including five cases of hepatitis B and one case of hepatitis C), but the virus copy was normal in these six patients. Patients who were transfusion-dependent (≥2 RBC units/month for ≥6 months) were expected to survive for >1 year after receiving a treatment of deferoxamine or deferasirox iron chelation therapy (ICT). The course of treatment last for 14 days. Among a total of 55 patients who had received ICT treatment, 46 patients had received the treatment of ICT for more than four courses, 38 had completed eight courses, 25 had completed 10 courses, and 18 had completed 16 courses. All patients underwent at least one MRI or DECT examination. The detection time was before ICT or 6 months after ICT. Sixty-three patients underwent DECT. The VIC liver and cardiac values were expressed in mg/ml. One-hundred-and-forty-one patients underwent 3.0-T MRI. There was no intolerance during the MRI examination. The T2^*^ values were converted into LIC and MIC, expressed in mg/g. Twenty-three patients underwent DECT and MRI examination on the same day. The data of Serum ferritin (SF) and CRP was collected on the same day of DECT/MRI examination. In the MRI group, 91% had liver IO. In the DECT group, 85% had liver IO. The clinical characteristics of the patients are shown in Table 1.

**Table 1:** Patient characteristics.

**Patient's characteristics**	**MRI group (*n* = 141)**	**DECT group (*n* = 63)**
Male/female	86/55	41/22
Age (m) (range)	55.58 (20–82)	52.77 (21–82)
**WHO classification, n(%)**		
MDS-SLD	2 (1.4%)	0 (0%)
MDS-MLD	83 (58.9%)	38 (60.3%)
MDS-RS	16 (11.3%)	15 (23.8%)
MDS-EB-1	19 (13.5%)	6 (9.5%)
MDS-EB-2	10 (7.1%)	3 (4.8%)
tAML	11 (7.8%)	1 (1.6%)
**IPSS-R risk, n (%)**		
Very low	6 (4.3%)	1 (1.6%)
Low	45 (31.9%)	24 (38.1%)
Intermediate	69 (48.9%)	33 (52.4%)
High	17 (12.1%)	4 (6.3%)
Very high	4 (2.8%)	1 (1.6%)
**WPSS risk, n (%)**		
Very low	10 (7.1%)	3 (4.8%)
Low	35 (24.8%)	16 (25.4%)
Intermediate	59 (41.8%)	29 (46.0%)
High	31 (22.0%)	13 (20.6%)
Very high	6 (4.3%)	2 (3.2%)
ASF(m) (range)	2106.65 (232–10,520)	1978.27 (232–6,774)
TS(m) (range)	80.06 (17.5–100)	73.46 (17.5–99)
Liver iron (m) (range)	LIC (mg/g)	Liver VIC (mg/ml)
ASF <1,000 ng/ml	6.4 (1.1–25)	0.67(*-2.49)
ASF ≥ 1,000 ng/ml	14.3 (2.6–69.6)	3.03 (0.83–8.76)
Cardiac iron (m) (range)	MIC (mg/g)	Cardiac VIC (mg/ml)
ASF <1,000 ng/ml	1.1 (0–5.6)	0.1 (*-2.5)
ASF ≥ 1,000 ng/ml	1.8 (0.4–11.7)	1.07 (*-6.83)
Transfusion dependence, n(%)	54 (38.3%)	23 (36.5%)
EPO(m) (range) (mIU/ml)	2,395 (3.7–24,753)	2,087 (10.5–19,269)
ALT (U/l)	20 (5–126)	36 (8–156)
BNP (ng/l)	155 (5–2284)	95.6 (28–1845.8)

m, Median; *, No iron deposition was detected; MDS, myelodysplastic syndrome; WHO, World Health Organization. The risk classification is determined according to the numerical rating system at the time of initial diagnosis. MDS-SLD, single lineage dysplasia; MDS-MLD, multi lineage dysplasia; MDS-RS, ring sideroblasts; MDS-EB, excess blasts; tAML, MDS transformed acute myeloid leukemia; IPSS-R, Revised International prognostic scoring system; WPSS, WHO classification-based prognostic scoring system; ASF, adjusted serum ferritin; TS, transferrin saturation. Transfusion-dependent: at least 4U of RBC with 8 weeks for hemoglobin <6 g/dL.

The clinical LIC grading thresholds were 1.8, 3.2, 7.0, and 15.0 mg Fe/g dry tissue (13). For the four clinical LIC thresholds, the corresponding cutoff values of VIC were 19.6, 25.3, 36.9, and 61.5 HU, respectively (14). The corresponding Iodine related attenuation measurements were 19.6, 25.3, 36.9, and 61.5 HU of quantification measurements. VIC were roughly 0.8, 1.1, 1.6, and 2.4 mg/ml (15). The result of X-ray spectral analysis of Fe(NO3)3 revealed a lower slope of CT numbers for iron compared with iodine (1.9 vs. 3.2) (16). Therefore, the iron VIC corresponding to iodine VIC 0.8, 1.1, 1.6, and 2.4 mg/ml were 1.34, 1.85, 2.69, and 4.03 mg/ml, respectively. In the MRI group, we defined LIC <1.8 (mg Fe/g) as the normal group, 1.8 ≤ LIC <3.2 (mg Fe/g) as the mild IO group, 3.2 ≤ LIC <7.0 (mg Fe/g) as the moderate IO group, 7.0 ≤ LIC <15.0 (mg Fe/g) as the severe IO group, and (mg Fe/g) 15.0 ≤ LIC as the extremely severe IO group. In the DECT group, we defined LVIC <1.34 (mg/ml) as the normal group, 1.34 ≤ LVIC <1.85 (mg/ml) as the mild IO group, 1.85 ≤ LVIC <2.69 (mg/ml) as the moderate IO group, 2.69 ≤ LVIC <4.03 (mg/ml) as the severe IO group, and 4.03 (mg/ml) ≤ VIC as the extremely severe IO group. According to the literature (17), the ASF <500 ng/ml is classified as the normal group, 500 ng/ml ≤ ASF <1,000 ng/ml as the mild IO group, 1,000 ng/ml ≤ ASF <2,500 ng/ml as the moderate IO group, 2,500 ng/ml ≤ ASF <5,000 ng/ml as the severe IO group, and 5,000 ng/ml ≤ ASF is classified as the extremely severe IO group.

### Iron Content in Liver and Cardiac Was Detected by MRI

**Figure 1:**
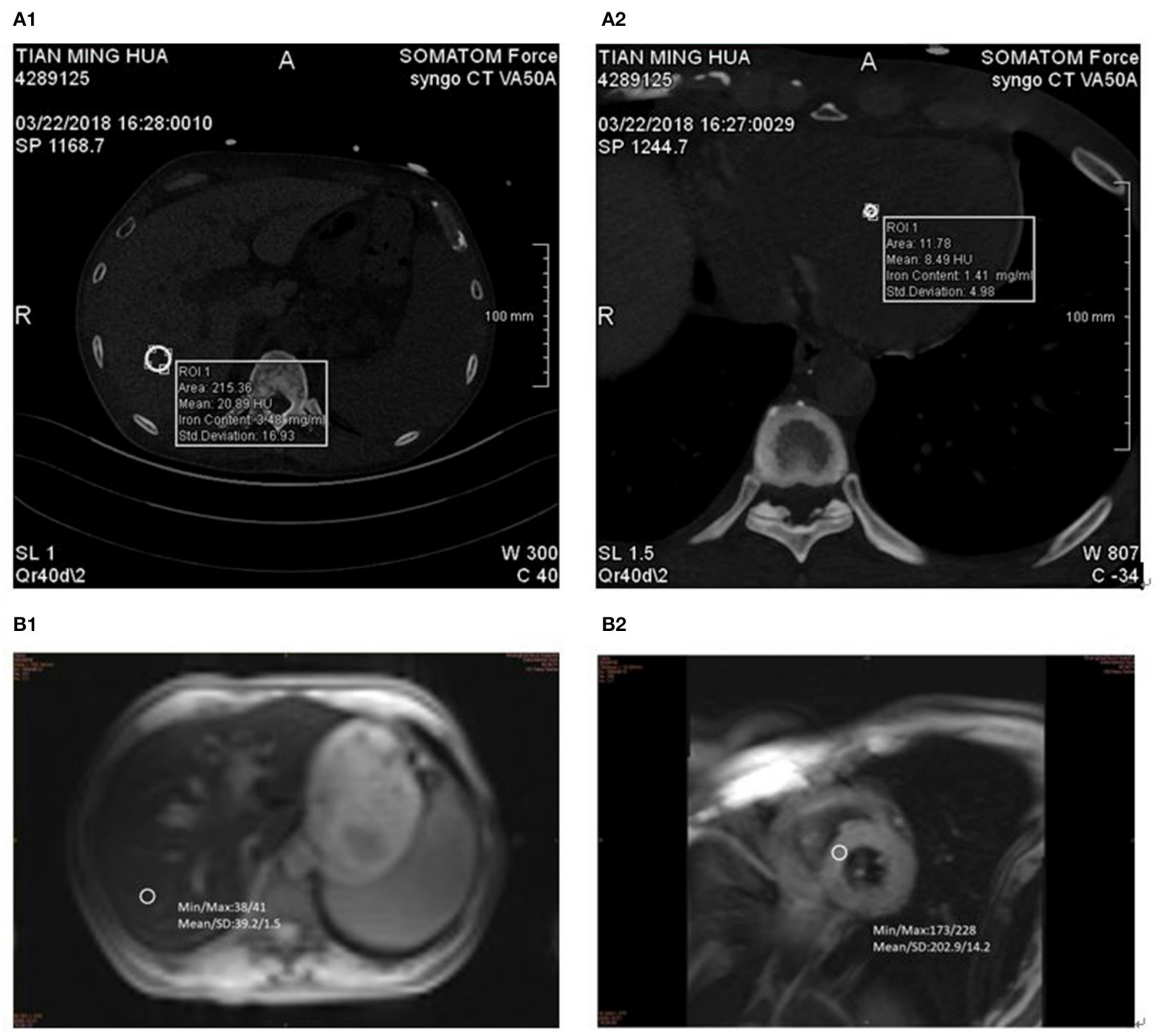
DECT and MR measurements in 57-year-old man with MDS-RS. ASF 5,807 ng/ml. **(A1)** liver VIC 4.67 mg/ml and **(A2)** cardiac VIC 6.83 mg/ml. **(B1)** LIC 11.2 mg/g and **(B2)** MIC 7.2 mg/g.

The MRI images (Siemens MAGNETOM Verio 3.0T) of liver and cardiac were collected by skilled operators. The routine abdominal scan included fat suppression T2 ^*^ WI in coronal and axial, dual echo T1 ^*^ WI in the same or opposite phase, and the diffusion weighted imaging (DWI) sequence. After routine scanning, a gradient-echo type multi-echo sequence was used to collect liver axial multi echo T2 ^*^ WI, and patients were asked to hold their breath at the end of each breath. Imaging parameters were as follows: echo time (TE) (2.0–11.8) ms, echo interval 0.6 MS, a total of 16 echoes, repetition time (TR) 200 ms, layer thickness of 10 mm, scanning time of 17 s (Figure 1B1). Then, T2 ^*^ myocardial imaging was performed. Two chamber and four chamber images were collected in turn. At the four-chamber heart level, the short axis position of the heart was collected at the place with the largest transverse diameter of the interventricular septum. A multi echo gradient echo sequence was used to instruct patients to hold their breath at the end of each breath. Imaging parameters: te (2.0–19.4) ms, echo interval 2.5 ms, total of 8 echoes, TR 22 ms, imaging matrix 256 × 128, slice thickness 10 mm, scanning time 12 s (Figure 1B2).

### Detection of Iron VIC in Liver and Cardiac by DECT

The same 2 × 192-layer DECT scanner (Siemens SOMATOM Force) was used for all examinations. The detection protocol used dual energy (De, 100 and 150 kvp for liver, 90 and 150 kvp for cardiac) with a thin filter to improve the separation of the two spectra. The image data was processed with the software prototype (DE IronVNC; syngo.via Frontier; Siemens Healthineers). The liver map was made by decomposing the basic materials into air, water, and iron. Skilled CT diagnosis radiologists performed the image analysis. The VIC of the liver was measured by placing three large freehand ROIs at the hepatic vault and about 1 cm below the hepatic capsule, excluding the larger vessels near the portal vein and inferior vena cava. Moreover, ROIs included the left lobe and the anterior part of the right lobe (Figure 1A1). The VIC of the cardiac was measured by placing three large freehand ROIs approximately at the mid-level of the heart (Figure 1A2). VIC in mg / ml was then obtained from ROI.

### Detection of Iron Metabolism Index

SF, brain natriuretic peptide (BNP), and erythropoietin (EPO) were determined by radioimmunoassay. Serum iron and total iron binding capacity were detected by ferrous nitrogen colorimetry. Alanine aminotransferase (ALT) was detected by pyruvate oxidase method. C-reactive protein (CRP) was detected by immunoturbidimetry. When CRP > 10 mg/L, ASF = SF/log 10 CRP; when CRP ≤ 10 mg/L, ASF = SF.

### Statistical Analysis

Data were analyzed using SPSS 17.0. Receiver operating characteristic (ROC) analysis was applied in all patients who underwent both DECT and 3.0T MRI or one of them. The significance of differences in the area under the ROC curve (Az) (two estimated binormal curves) of LVIC and LIC in different subgroups were assessed by the univariates-score test for comparison between the two modalities. When LVIC criterion at DECT and LIC criterion at MRI for clinically mild, moderate, severe, and extremely severe liver iron accumulation were set, sensitivity and specificity were performed. Numerical variables were expressed as mean ± standard deviation (SD), and non-numerical variables were expressed as frequencies or percentages. The Shapiro Wilk test was used to test the normal distribution of the parameters. One way ANOVA was used to compare the four groups. The LSD-t method was used to compare these two groups, and an independent sample *t*-test was used to compare these two groups for numerical comparison, and a chi square test was used for a non-numerical comparison. The Spearman correlation analysis was used to analyze the correlation between these two groups. *p* <0.05 was considered statistically significant.

## Results

**Figure 2:**
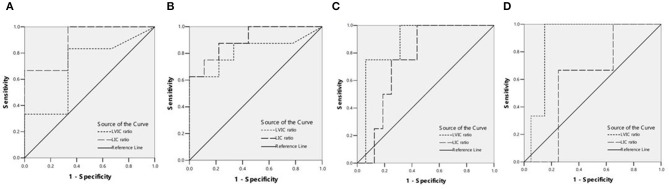
**(A)** ROC curves showed the comparison of liver VIC (LVIC) and LIC in differentiating patients with ASF < 500 ng/ml or 500 = ASF < 1,000 ng/ml (9 patients). LIC Az was 0.889, and LIC at 1.8 (mg Fe/g) sensitivity and specificity were 100 and 33.3%, respectively. LVIC Az was 0.694, and LVIC at 1.34 (mg/ml) sensitivity and specificity were 33.3 and 66.7%, respectively. There was significant difference between LVIC and LIC sequences (*p* < 0.05). **(B)** ROC curves showed the comparison of LVIC and LIC in differentiating patients with ASF < 1,000 ng/ml or 1,000 = ASF < 2,500 ng/ml (17 patients). LIC Az was 0.903, and LIC at 3.2 (mg Fe/g) sensitivity and specificity were 100 and 33.3%, respectively. LVIC Az was 0.819, and LVIC at 1.85 (mg/ml) sensitivity and specificity were 62.5 and 88.9%, respectively. There was no significant difference between LVIC and LIC sequences (*p* > 0.05). **(C)** ROC curves showed the comparison of LVIC and LIC in differentiating patients with ASF < 2,500 ng/ml or 2,500 = ASF < 5,000 ng/ml (20 patients). LIC Az was 0.750, and LIC at 7.0 (mg Fe/g) sensitivity and specificity were 75 and 56.2%, respectively. LVIC Az was 0.875, and LVIC at 2.69 (mg/ml) sensitivity and specificity were 75 and 81.2%, respectively. There was no significant difference between LVIC and LIC sequences (*p* > 0.05). **(D)** ROC curves showed the comparison of LVIC and LIC in differentiating patients with ASF < 5,000 ng/ml or 5,000 = ASF ng/ml (23 patients). LIC Az was 0.617, and LIC at 15.0 (mg Fe/g) sensitivity and specificity were 100 and 30%, respectively. LVIC Az was 0.883, and LVIC at 4.03 (mg/ml) sensitivity and specificity were 100 and 85%, respectively. There was significant difference between LVIC and LIC sequences (*p* < 0.05).

**Figure 3:**
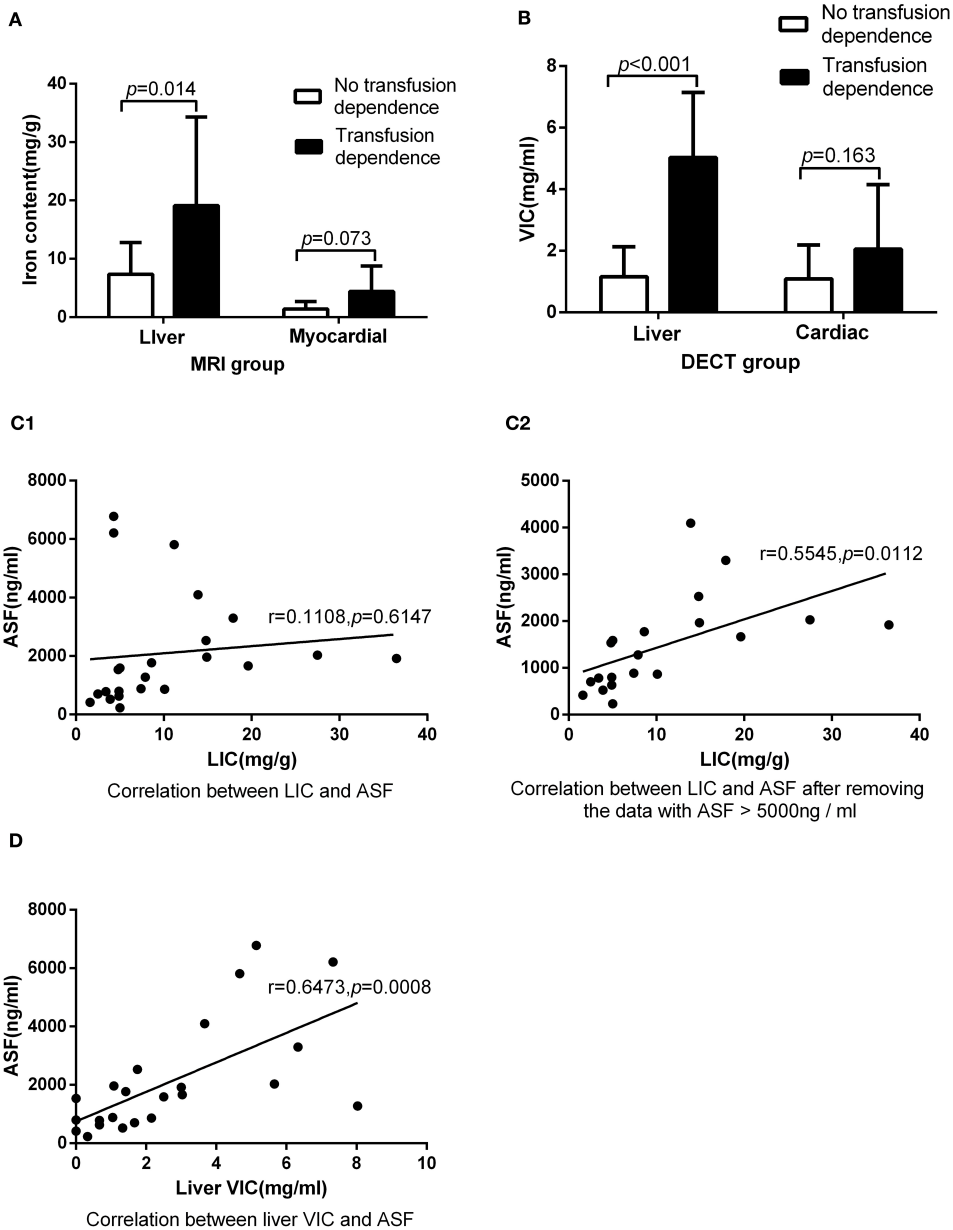
Underwent DECT and MRI examination on the same day: **(A)** In MRI group, there was significant difference between patients with transfusion dependence and without transfusion dependence (19.12 ± 15.17 vs. 7.34 ± 5.43, *p* < 0.05) in LIC, but there was no significant difference between them (4.40 ± 4.36 vs. 1.40 ± 1.27, *p* > 0.05) in MIC. **(B)** In DECT group, there was significant difference between patients with transfusion dependence and without transfusion dependence (5.03 ± 2.11 vs. 1.16 ± 0.97, *p* < 0.05) in liver VIC, but there was no significant difference between them (2.09 ± 2.06 vs. 1.10 ± 1.09, *p* > 0.05) in cardiac VIC. **(C1)** LIC was not correlated with ASF (*r* = 0.11, *p* > 0.05). **(C2)** LIC was correlated with ASF after the exclusion of three ASF > 5,000 ng/ml corresponding data (*r* = 0.55, *p* < 0.05). **(D)** Liver VIC was correlated with ASF (*r* = 0.65, *p* < 0.05).

**Figure 4:**
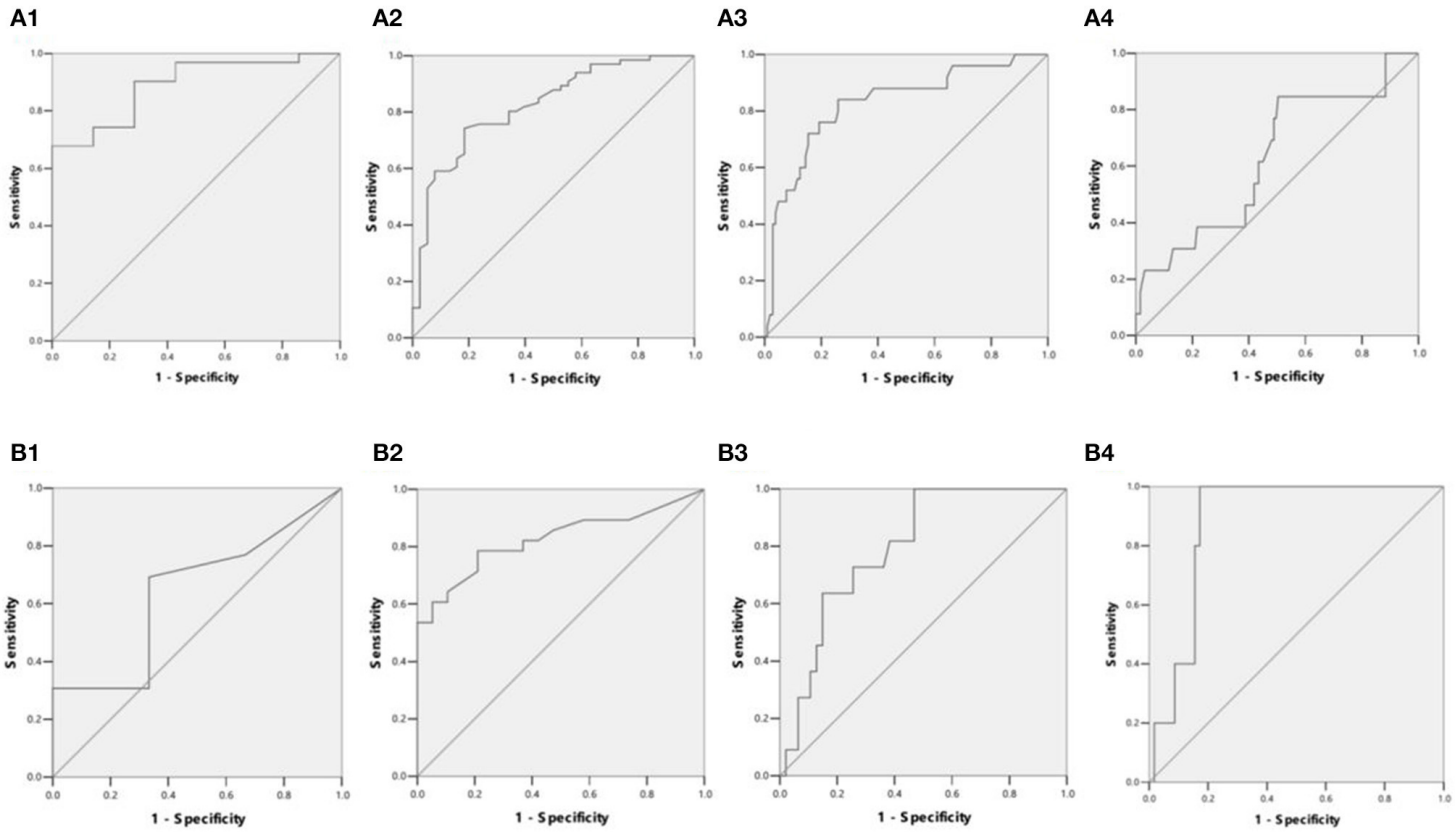
**(A1)** ROC curves showed LIC in differentiating patients with ASF < 500 ng/ml or 500 ≤ ASF < 1,000 ng/ml (38 patients). LIC Az was 0.889, and LIC at 1.8 (mg Fe/g) sensitivity and specificity were 96.8 and 42.9%, respectively. **(A2)** ROC curves showed LIC in differentiating patients with ASF < 1,000 ng/ml or 1,000 ≤ ASF < 2,500 ng/ml (104 patients). LIC Az was 0.824, and LIC at 3.2 (mg Fe/g) sensitivity and specificity were 98.5 and 21.1%, respectively. **(A3)** ROC curves showed LIC in differentiating patients with ASF < 2,500 ng/ml or 2,500 ≤ ASF < 5,000 ng/ml (129 patients). LIC Az was 0.826, and LIC at 7.0 (mg Fe/g) sensitivity and specificity were 88 and 36.5%, respectively. **(A4)** ROC curves showed LIC in differentiating patients with ASF < 5,000 ng/ml or 5,000 ≤ ASF ng/ml (141 patients). LIC Az was 0.628, and LIC at 15.0 (mg Fe/g) sensitivity and specificity were 38.5 and 65.1%, respectively. **(B1)** ROC curves showed liver VIC (LVIC) in differentiating patients with ASF < 500 ng/ml or 500 ≤ ASF < 1,000 ng/ml (19 patients). LVIC Az was 0.641, and LVIC at 1.34 (mg/ml) sensitivity and specificity were 30.8 and 83.3%, respectively. **(B2)** ROC curves showed LVIC in differentiating patients with ASF < 1,000 ng/ml or 1,000 ≤ ASF < 2,500 ng/ml (47 patients). LVIC Az was 0.825, and LVIC at 1.85 (mg/ml) sensitivity and specificity were 67.9 and 84.2%, respectively. **(B3)** ROC curves showed LVIC in differentiating patients with ASF < 2,500 ng/ml or 2,500 ≤ ASF < 5,000 ng/ml (58 patients). LVIC Az was 0.796, and LVIC at 2.69 (mg/ml) sensitivity and specificity were 72.7 and 74.5%, respectively. **(B4)** ROC curves showed LVIC in differentiating patients with ASF < 5,000 ng/ml or 5,000 ≤ ASF ng/ml (63 patients). LVIC Az was 0.883, and LVIC at 4.03 (mg/ml) sensitivity and specificity were 100 and 82.8%, respectively.

**Figure 5:**
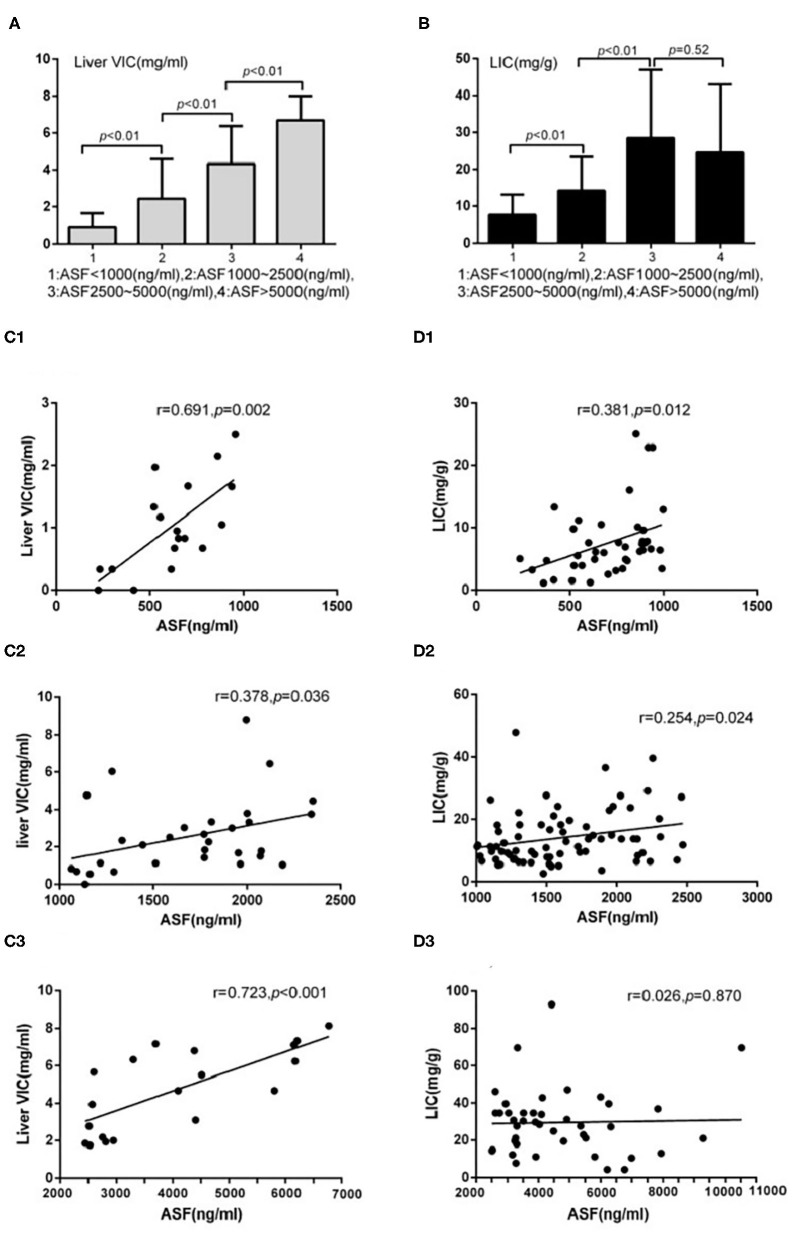
Underwent DECT/MRI examination: **(A)** Significant difference exists between the subgroups with ASF < 1,000 and 1,000 ≤ ASF < 2,500 subgroups in liver VIC expression (0.88 ± 0.77 vs. 2.44 ± 2.14, *p* < 0.05). Significant difference exists between the subgroups with 1,000 ≤ ASF < 2,500 and 2,500 ≤ ASF < 5,000 (2.44 ± 2.14 vs. 4.33 ± 2.04, *p* < 0.05). Significant difference between the subgroups with 2,500 ≤ ASF < 5,000 and 5,000 ≤ ASF subgroups (4.33 ± 2.04 vs. 6.69 ± 1.32, *p* < 0.05). **(B)** Significant difference exists between the subgroups with ASF < 1,000 and 1,000 ≤ ASF < 2,500 in LIC expression (7.60 ± 5.52 vs. 14.20 ± 9.17, *p* < 0.05). Significant difference exists between the subgroups with 1,000 ≤ ASF < 2,500 and 2,500 ≤ ASF < 5,000 in LIC expression (14.20 ± 9.17 vs. 28.45 ± 18.55, *p* < 0.05). No significant difference exists between the subgroups with 2,500 ≤ ASF < 5,000 and 5,000 ≤ ASF in LIC expressions (28.45 ± 18.55 vs. 24.46 ± 18.53, *p* < 0.05). **(C1)** There were significant correlation between Liver VIC and ASF in the subgroup with ASF < 1,000 ng/ml (*r* = 0.69, *p* < 0.05). **(C2)** There were significant correlation between Liver VIC and ASF in the subgroup with 1,000 ≤ ASF < 2,500 ng/ml subgroup (*r* = 0.38, *p* < 0.05). **(C3)** There were significant correlation between Liver VIC and ASF in the subgroup with 2,500 ng/ml ≤ ASF (*r* = 0.72, *p* < 0.05). **(D1)** There were significant correlation between LIC and ASF in the subgroup with ASF < 1,000 ng/ml (*r* = 0.38, *p* < 0.05). **(D2)** There were significant correlation between LIC and ASF in the subgroup with 1,000 ≤ ASF < 2,500 ng/ml (*r* = 0.25, *p* < 0.05). **(D3)** There were no significant correlation between LIC and ASF in the subgroup with 2,500 ng/ml ≤ ASF subgroup (*r* = 0.03, *p* > 0.05).

The detection rates of liver IO by MRI and DECT in different subgroups of ASF are shown in Table 2. The ROC curves of liver iron content on DECT and MRI for patients who underwent DECT and MRI examination on the same day are shown in Figure 2, and the correlation results of LIC and liver VIC with ASF, and liver and cardiac iron expression in transfusion dependent and non-transfusion dependent patients are shown in Figure 3. Moreover, the ROC curves of liver iron content on DECT or MRI are shown in Figure 4. LIC and LVIC expression of the different subgroups in ASF, the correlation between LIC and ASF, and the correlation between LVIC and ASF in each subgroup are shown in Figure 5. Further analysis showed that there was no significant correlation between LIC and ASF in the subgroup with 2,500 ≤ ASF <5,000 ng/ml (*r* = 0.227, *p* = 0.245), and no significant correlation between LIC and ASF in the subgroup with 5,000 ng/ml ≤ ASF (*r* = 0.447, *p* = 0.110).

**Table 2:** Detection rate of liver iron concentration by MRI and DECT in ASF subgroups.

**ASF(ng/ml)**	**MRI group**				**DECT group**			
	**Normal**	**Mild **~** Moderate**	**Severe**	**Extremely severe**	**Normal**	**Mild **~** Moderate**	**Severe**	**Extremely severe**
**Group 1**								
<1,000	0/9	7/9	2/9	0/9	8/9	0/9	1/9	0/9
1,000–2,500	0/8	2/8	2/8	4/8	2/8	1/8	3/8	2/8
2,500–5,000	0/3	1/3	1/3	1/3	0/3	1/3	1/3	1/3
5,000 ≤	0/3	2/3	1/3	0/3	0/3	0/3	0/3	3/3
**Groups 2**								
<1,000	4/38	18/38	12/38	4/38	13/18	3/18	2/18	0/18
1,000–2,500	0/66	13/66	32/66	21/66	5/28	5/28	12/28	6/28
2,500–5,000	0/25	3/25	5/25	17/25	1/11	1/11	4/11	5/11
5,000 ≤	0/12	3/12	4/12	5/12	0/5	0/5	1/5	4/5
**Groups 3**								
<1,000	2/21	14/21	5/21	0/21	16/17	1/17	0/17	0/17
1,000 ≤	0/12	1/12	9/12	2/12	6/8	1/8	1/8	0/8

*Group 1. Patients who underwent DECT and MRI examination at the same time. Group 2. Patients who underwent DECT or MRI examination not at the same time. Group 3. Patients who not received RBC transfusion.*

The analysis of myocardial iron content showed that there was no significant correlation between cardiac VIC and ASF in the subgroup with ASF <1,000 ng/ml (*r* = 0.253, *p* = 0.268), 1,000 ≤ ASF <2,500 ng/ml (*r* = 0.098, *p* = 0.575), and 2,500 ≤ ASF ng/ml subgroup (*r* = 0.435, *p* = 0.055). There was also no significant correlation between MIC and ASF in the subgroup with ASF <1,000 ng/ml (*r* = 0.090, *p* = 0.570), 1,000 ≤ ASF <2,500 ng/ml (*r* = 0.135, *p* = 0.232), and 2,500 ≤ ASF ng/ml (*r* = 0.083, *p* = 0.596).

## Discussion

MRI and DECT are available as the present non-invasive standard practice in the diagnosis of iron overload diseases. The results of our study demonstrate that the area under the ROC curve (Az) of LVIC in the DECT group with ASF <1,000 ng/ml was significantly lower than LIC in the 3.0T MRI group with ASF <1,000 ng/ml. In the subgroup with 1,000 < ASF ≤ 2,500 and 2,500 < ASF ≤ 5,000, there was no significant difference between LVIC and LIC in the area under the ROC curve (Az). However, in the subgroup with 5,000 ng/ml ≤ ASF, the area under the ROC curve (Az) of LVIC in the DECT group was significantly higher than that of LIC in the MRI group. These findings were consistent, regardless of whether the patients underwent DECT, MRI, or both on the same day. These results show that DECT is not sensitive to assessing the low iron content and 3.0T MRI is not accurate in patients with high iron content. The analysis of subgroups in patients undergoing DECT/MRI showed that no difference exits between the subgroups with ASF 2,500–5,000 ng/ml and 5,000 ng/ml ≤ ASF in patients who underwent LIC, while a significant difference is observed between these two subgroups in patients who underwent liver VIC. A correlation analysis in the patients who underwent DECT and MRI on the same day shows that LVIC is positively correlated with ASF but LIC is uncorrelated with ASF. Moreover, LIC is only positively correlated in patients with ASF <5,000 ng/ml. Further, a large sample shows that LIC is correlated with ASF but only in patients with ASF <2,500 ng/ml, while LIC is not correlated with ASF in patients with 2,500 ng/ml ≤ ASF. Therefore, we can conclude that the measurement of LIC may be deviated in patients with ASF > 2,500 ng/ml. The deviation is particularly obvious in patients with ASF > 5,000 ng/ml, while the assessment of liver VIC remains accurate.

LIC can be employed to examine the iron load state of the whole body. However, our study shows that the accuracy of DECT in the detection of liver iron is not as high as that of 3.0TMRI in patients with ASF <1,000 ng/ml, and the accuracy of 3.0T MRI in the detection of liver iron is not as high as that of DECT in patients with 5,000 ng/ml ≤ ASF. In patients with SF 1,000–2,500 ng/ml, there is no significant difference between 3.0T MRI and DECT but in the patients with ASF 2,500–5,000 ng/ml, the LIC may be partly deviated.

At present, 1.5T and 3.0T MRI have been used for iron content detection in the clinic. However, 1.5T MRI is incapable of assessing heavy iron concentration, e.g., LIC>30 mg iron per gram of dry tissue. Extremely rapid signal decay may limit accurate liver iron quantification (2). Clinically, 1.5T is gradually replaced by 3.0T MRI. While 3.0T imaging provides higher signal to noise than 1.5T, susceptibility artifacts are worse than at 1.5T, which may degrade grade echo image quality. More importantly, due to faster signal decay at 3.0T, the maximum quantification limit may be lower than at 1.5T (18). The disadvantage of DECT is that VIC is not sufficiently sensitive to detecting normal iron levels, since the calibration of the dual-energy algorithm for VIC imaging is obtained from an *ex vivo* phantom study, which might not be optimal for patients. This factor might explain why some negative VIC values appeared in several patients with normal and mild iron accumulation (5). Furthermore, a liver biopsy is used as the standard to compare the hepatic iron content detection of 3.0T MRI and DECT in patients with hepatitis and liver transplantation. The results show that the diagnostic performance of DECT is similar to that of MRI, while DECT is not as accurate as MRI in detecting and quantifying low level iron content (19).

There is no significant correlation between cardiac VIC and ASF, and MIC and ASF in all subgroups with different levels of ASF. Moreover, the estimation of the liver iron concentration in the transfusion dependent group is higher than that in the non-transfusion dependent group, with the employment of DECT and MRI. However, the cardiac iron concentration has no significant difference between the employment of DECT and MRI. Since more than 70% of iron is deposited in the liver (20), and cardiac iron deposition is relatively slight compared with liver, cardiac iron content cannot reflect the real iron content in the body.

Among the non-transfusion MDS patients detected by 3.0T MRI, 84.8% have hepatic iron overload, while 39.4% have moderate to severe hepatic iron overload. The results may be related to the ineffective hematopoiesis and abnormal iron metabolism in MDS patients. However, only three patients detected by DECT have liver iron overload. This may be the result of the poor sensitivity to lower iron concentrations. For MDS patients without RBC transfusion but combined with moderate to severe liver IO, knowing whether ICT is necessary is worth studying further.

Our study had several limitations. First, the standard of reference analysis is ASF instead of liver biopsy. Most MDS patients are associated with thrombocytopenia. For safety reasons, we did not perform liver biopsy. Second, since the number of cases that are simultaneously performed by DECT and MRI is small, there may be selection bias between MRI and DECT in different groups.

This study shows that MRI and DECT are complementary to each other. In patients with high iron content, such as ASF ≥ 5,000 ng/ml, the results of DECT in the detection of LVIC is more reliable. In patients with low iron content, such as ASF <1,000 ng/ml, the results of MRI in the detection of LIC is more reliable. For the sake of more accurately evaluating iron content in MDS patients, the appropriate detection method can be selected according to the level of ASF in patients.

## Data Availability Statement

The original contributions presented in the study are included in the article/supplementary material, further inquiries can be directed to the corresponding author/s.

## Ethics Statement

Written informed consent was obtained from the individual(s) for the publication of any potentially identifiable images or data included in this article.

## Author Contributions

YZ: experimental design, statistical analysis, and thesis writing. CX: collection and arrangement of experimental data. JL: instrument operation. L-xS, Y-sZ, SH, Z-wL, and CG: assist to collect and arrange experimental data. J-gZ: guide instrument data analysis. C-kC: provide the overall idea and thesis modification. All authors contributed to the article and approved the submitted version.

## Conflict of Interest

The authors declare that the research was conducted in the absence of any commercial or financial relationships that could be construed as a potential conflict of interest.
